# Real-time energy-saving metro train rescheduling with primary delay identification

**DOI:** 10.1371/journal.pone.0192792

**Published:** 2018-02-23

**Authors:** Hangfei Huang, Keping Li, Paul Schonfeld

**Affiliations:** 1 State Key Laboratory of Rail Traffic Control and Safety, Beijing Jiaotong University, Beijing, China; 2 Department of Civil and Environmental Engineering, University of Maryland, College Park, Maryland, United States of America; Beihang University, CHINA

## Abstract

This paper aims to reschedule online metro trains in delay scenarios. A graph representation and a mixed integer programming model are proposed to formulate the optimization problem. The solution approach is a two-stage optimization method. In the first stage, based on a proposed train state graph and system analysis, the primary and flow-on delays are specifically analyzed and identified with a critical path algorithm. For the second stage a hybrid genetic algorithm is designed to optimize the schedule, with the delay identification results as input. Then, based on the infrastructure data of Beijing Subway Line 4 of China, case studies are presented to demonstrate the effectiveness and efficiency of the solution approach. The results show that the algorithm can quickly and accurately identify primary delays among different types of delays. The economic cost of energy consumption and total delay is considerably reduced (by more than 10% in each case). The computation time of the Hybrid-GA is low enough for rescheduling online. Sensitivity analyses further demonstrate that the proposed approach can be used as a decision-making support tool for operators.

## Introduction

A train timetable is an operation plan for scheduling trains with preset times and orders. The train timetable optimization problem is also known as the train scheduling/rescheduling problem. While the expanding coverage scale of metro networks brings great convenience to residents, it also raises some problems yet to be tackled in terms of train controls and traffic management (Ma et al. [1]). One of the most pervasive problems is train delays, for which specific response methods are needed. For example, on a metro line, if one train is delayed at a station, its succeeding trains are affected in many ways. The typical situation is: the next train has to decelerate or even stop in the middle of the section, so that the delay is propagated and the travel time is prolonged. After the delayed train departs, the next train must accelerate again to catch up with the timetable, thereby consuming more energy. From a system perspective, dealing with the original delay of the preceding train is often the key factor in reducing the propagated delays, thereby alleviating their effects on the system. In a rail transit system, such original delays are called primary delays, and delays in succeeding trains caused by them are called flow-on delays. In a delay scenario, the original timetable is disrupted, and trains must be rescheduled. The delay is one of the disturbances that often occur in a rail transit system, and the aim of train rescheduling is to change the ideal timetable as little as possible, while satisfying a set of operational constraints.

The train timetable scheduling problem is addressed at two levels: the macro-level and the micro-level. The macro-level studies mainly focus on the evaluation of system performance, and the problem is often formulated with multi-objective models (Xu et al. [2]; Ma et al. [3]), sometimes considering fuzzy variables (Yang et al. [4]). The objectives include optimizing train travel time (Chevrier et al. [5]; Robenek et al. [6]), minimizing energy consumption (Yang et al. [7]; Yin et al. [8]), predicting passenger flow and ridership (Ding et al. [9]; Li et al. [10]), and reducing passenger travel and wait time (Wang et al. [11]; Robenek et al. [12]). Since multi-objective models are mostly considered NP-hard and nonlinear, the optimization solutions mainly consist of heuristic algorithms (Zhou et al. [13]) and relaxation techniques (Liu et al. [14]). Most macro-level studies only optimize train arrivals/departures at stations, and do not consider the characteristics of train movements. Thus, in these works the train speed profiles are considered ideal. However, different line conditions (e.g., grades, layouts) correspond to different ideal speed profiles. In this regard, the micro-level studies mainly focus on obtaining the ideal speed profiles under different line conditions. For example, Howlett et al. [15] and Albrecht et al. [16] try to optimize the speed profile by analytical methods to calculate optimal switching points for train controls. Since the micro-level studies usually have only one objective, i.e., reducing energy, many other studies try to integrate the problems in both levels. They first generate the ideal speed profile in each section with a given trip time, and then jointly optimize the train timetable (Li and Lo [17]). Some of these studies also account for regenerative braking to synergistically schedule adjacent trains (Su et al. [18]; Sicre et al. [19]), thereby saving energy from braking. In general, the train scheduling problem has received considerable attention, and many models and solution approaches are effective for scheduling trains. However, due to the complexity of the models that require hundreds of decision variables in large-scale networks, the computation cost remains high. It is still a big challenge to reduce the scale of decision variables. Moreover, many timetable optimization methods cannot predict future traffic speed (Ma et al. [20]) and do not consider the potential conflicts among trains. In practical operation, if there are train interactions occurring due to disturbances, the original ideal speed profiles cannot be achieved by the affected trains, and thus the obtained timetable is no longer practical. Therefore, train interactions should be considered for more practical solutions.

To account for different types of train interactions, simulation methods can provide alternative solution. There are a few existing simulation methods that consider train interactions. Specifically, based on the train advance strategy (Dorfman and Medanic [21]), Li et al. [22] extend the algorithm to consider train movement characteristics, and use a branch and bound algorithm to avoid future conflicts; Xu et al. [23] propose a simulation algorithm that defines the train acceleration, cruising and deceleration as discrete events, to simulate train movements on a metro line. In their studies, the preset section trip times determine the ideal speed profiles, thereby affecting energy consumption. However, these simulation methods do not provide an optimized solution in the delay scenarios. That is, train rescheduling methods should be integrated with simulation under delays.

Unlike the train scheduling problem, train rescheduling problem mainly focuses on disturbance management, to reduce the total delay and generate a conflict-free timetable (Yin et al. [24]). Much research related to the train rescheduling is based on the Alternative Graph model (Cacchiani et al. [25]). For example, D’ariano et al. [26] formulate the problem as a job shop scheduling model, and propose a branch and bound algorithm to reschedule the trains. D’ariano et al. [27] implement the alternative graph model in the real-time railway traffic management system ROMA (Railway Traffic Optimization by Means of Alternative Graphs). In the same testbed with ROMA, Corman et al. [28] and Corman et al. [29] consider different delay scenarios, and describe the graph-based solutions. However, the alternative graph cannot model train interactions, and thus a new graph model considering detailed train motions is worth investigating. Many other studies focusing on train delays use Mixed Integer Programming (MIP) model to formulate the rescheduling problem. For example, Tornquist and Persson [30] propose a MIP model considering train rerouting and reordering. Acuna et al. [31] extend their MIP model to formulate the railway rescheduling problem. To improve the computation efficiency, Tornquist [32] designs a heuristic greedy approach for the same problem. However, how to identify the affected trains and sections under disturbances is not clearly discussed in those studies.

In addition, big data techniques are essential for today’s rail transit systems (Ma et al. [33]). Particularly, analytic visualization and critical path algorithm are already implemented in train delay analysis. For example, the chromatic train diagram (Tomii [34]) intuitively displays different levels of delays with different colors on the train timetable. Yamamura et al. [35] use the critical path algorithms to analyze delay propagation, and visualize the results on train timetable for the first time. The critical path algorithm has advantages in the delay-propagation and conflict-identification fields, compared to other methods (Meester and Muns [36]; Daamen et al. [37]; Kecman and Goverde [38]). For example, Goverde [39] proposes the discrete time graph model to study the delay-propagation. With the critical path algorithm, different types of delays and delay behaviors are analyzed, including primary and secondary delays, structural delays, periodic delay regimes, and delay explosion. Therefore, the application of critical path algorithms in delay scenarios motivates the study of this paper.

In general, this paper considers the aforementioned gaps, and proposes a two-stage optimization method (the basic idea is similar to Wang et al. [40]). The first stage is a critical path algorithm which aims to identify the primary and flow-on delays, and the second stage is a hybrid genetic algorithm which reschedules the affected trains under those delays, with an optimized objective function. Besides, the analytic visualization and simulation method are also used here. The main contributions of this paper are highlighted below:

1. With given delays, a simulation method and a train status graph are used for presentation of expected flow-on delays and effects. Then, with analytic visualization methods, a critical path algorithm is designed to identify the primary and flow-on delays. The identification result is represented by critical paths. Each critical path consists of a primary delay, its flow-on effects and delays, and the sections affected by this delay, with corresponding time stamps. Since no other studies in the reviewed literature analyze the delay scenarios with both simulation and delay classification, this is a novel design in the train rescheduling field.
2. With the integration of the identification results and the hybrid genetic algorithm, the solution approach reschedules the affected trains with consideration of both system performance and train movement characteristics. In this way, more practical results are obtained. The identification results greatly reduce the scale of decision variables in the GA, so that the original timetable is less changed. The computation time of the algorithm is much lower than the generic one that accounts for all affected trains in their remaining journeys, thereby improving the computation efficiency. Despite smaller inputs, results show that the solution is optimized.

The rest of this paper is organized as follows. In Section 2, the specific problem is simplified with a graph, and formulated with a mixed integer model. In Section 3, the solution approach consisting of the primary delay identification algorithm and the hybrid-GA are proposed. In Section 4, the effectiveness and efficiency of the proposed approach are demonstrated in the actual Beijing Subway Line 4. In Section 5, the main contributions of this paper are summarized and some future research is discussed.

## Problem formulation and model presentation

### Notation description

The notation and decision variables throughout this paper are listed in Table 1.

**Table 1:** Notation and decision variables.

**Sets**	
*I*	Set of affected trains.
*K*	Set of affected stations.
*Z*	Set of affected sections.
*T*	Set of time stamps.
*U*	Set of train running information.
*D*	Set of identification result data.
**Notation**	
*i*	Train index, *i* ∈ *I*.
*k*	Station index for *k* ∈ *K*; and section index for *k* ∈ *Z*.
*t*	Time stamp, *t* ∈ *T*.
*$v_{i}^{k}(t)$*	Speed of train *i* in section *k* at time *t* (m/s).
$s_{i}^{k}(t)$	Position of train *i* in section *k* at time *t* (m).
$u_{i}^{k}(t)$	Acceleration/deceleration rate of train *i* in section *k* at time *t* (m/s^2^).
$F_{i}^{k}(t)$	Tractive effort of train *i* in section *k* at time *t* (N).
$fr(v_{i}^{k}(t))$	Resistance of train *i* in section *k* at time *t*, which is a function of train speed $v_{i}^{k}(t)$, based on the Davis Equation (N).
$P_{i}^{k}(t)$	Tractive power of train *i* in section *k* at time *t* (w/s).
*dis*	Distance between two adjacent trains (m).
*o*	Train control strategy (0 = accelerating, 1 = cruising, 2 = decelerating).
*m*_*i*_	Mass of train *i*, which is a constant along the line (Kg).
$E_{i}^{k}$	Energy consumed by train *i* in section *k* (J).
*E*_*total*_	Total energy consumed by all trains in a system (J).
$T_{delay}^{i}$	Delay for train *i* (s).
$T_{delay}^{total}$	Total delay of all trains (s).
*S*_*M*_	Required minimum distance (“safety margin”) for adjacent trains (m).
*χ*_*s*_	Safety coefficient considering train movement characteristics
*S*_*b*_	Braking distance for a train from current speed to complete stop (m)
*S*_*safe*_	Safe distance to ensure adjacent trains not break the “safety margin” (m)
$A_{i}^{k}$	Planned arrival time for train *i* at station *k* (s).
$\alpha_{i}^{k}$	Number of passengers getting off train *i* at station *k* (passengers).
$\beta_{i}^{k}$	Number of passengers getting onto train *i* at station *k* (passengers).
*Cap*^*k*^	Platform passenger capacity at station *k* (passengers).
$Q_{i}^{k}$	Cumulative passengers between the departure times of train *i* and train *i-1* (passengers)
$N_{i}^{k}$	Number of passengers at station *k* when train *i* arrives (passengers).
*λ*^*k*^	Passenger arrival rate at station *k* (passengers/s).
*η*^*k*^	Maximum deviation value (threshold) for the affected trains (s).
*h*_min_	Minimum headway (s).
$\left[\underline{\omega^{k}},\bar{\omega^{k}}\right]$	Dwell time threshold for the affected trains at station *k* (s).
$\left[\underline{t^{k}},\bar{t^{k}}\right]$	Trip time threshold in the section between station *k* and *k+1* (s).
*w*_*e*_	Weighting factor for energy.
*w*_*t*_	Weighting factor for delay.
*c*_*e*_	Average cost of energy (yuan/Kwh).
*c*_*t*_	Average cost of delay (yuan/hour).
*C*_*total*_	Total cost of the system (yuan).
*z*	Index of train running record (in the data set *D*).
**Decision variable**	
$a_{i}^{k}$	Arrival time for the affected train *i* at station *k* (s).
$d_{i}^{k}$	Arrival time for the affected train *i* at station *k* (s).

### Problem description

**Fig 1:**
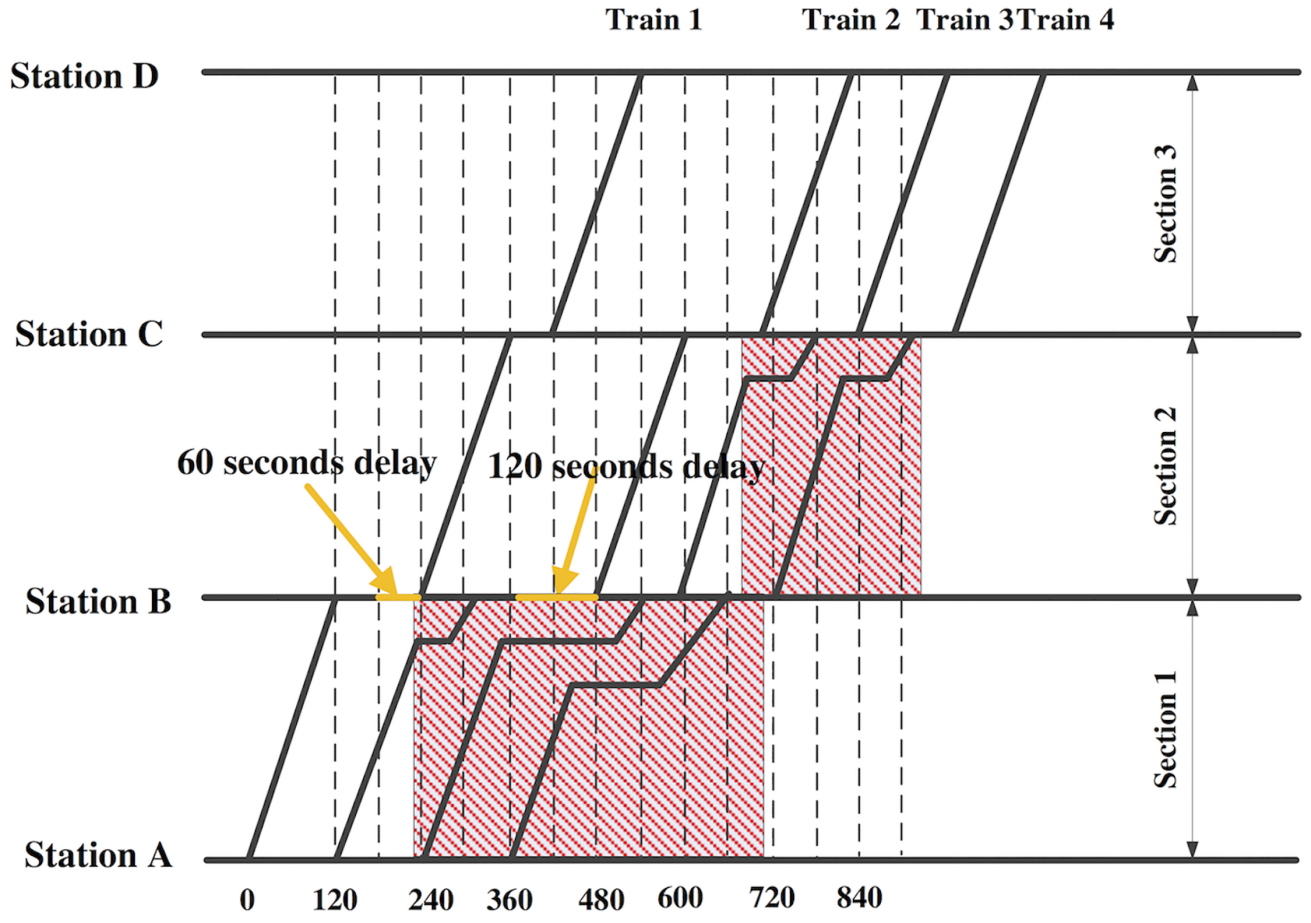
Primary delays and their flow-on effects in the timetable.

As shown in Fig 1, 4 trains are scheduled to travel on the line. At station B, at time 180s and 360s, train 1 is delayed for 60s, and train 2 is delayed for 120s. The primary delays cause the succeeding trains (train 2 and train 3) to wait in section 1, until the dwelling trains depart from station B. Although there is no primary delay found for train 3, train 4 is observed to have an unplanned stop in the middle of section. Such a phenomenon is considered as the propagated influence of the primary delay, and is called the flow-on delay. The flow-on delays can be also observed for train 3 and 4 in section 2. The affected zones of trains are shown in the shaded areas in section 1 and section 2. Moreover, it should be pointed that sometimes the delays cannot be detected at the very beginning. For example, in Fig 1, the flow-on delays may be detected at time 600s, when the first primary delay has already occurred for 420s. Therefore, the main focus of this paper is to reschedule the affected trains in their affected sections as soon as the primary delays are detected.

In addition, the energy consumption and the service level are two key factors in the metro system performance, and there are trade-offs between them. On one hand, with the same control strategy, longer section trip time reduces energy consumption; on the other hand, lower travel time improves service level, and reduces total travel delay. Moreover, in the peak hours, the headways between adjacent trains are very small. Without proper train rescheduling, even a small delay would severely influence the succeeding trains and the whole system. Thus, the train rescheduling should balance the energy and delay, while controlling delay propagation.

This paper studies the real-time train rescheduling problem in delay scenarios during peak hours, considering the conflicting objectives of energy and delay. To formulate the problem with a proper model and develop corresponding solution approach, following assumptions are made throughout the paper:

(A1) All trains on the metro line follow the FIFO rule. Each station can host only one train at a time, and no train overpassing is allowed throughout the line.
(A2) The disruptions (e.g. natural disasters, serious accidents) that would cause large-scale severe delays are not considered in this paper, because when such disruptions occur, the whole system might be shut down or emergency responses might be taken, which are outside the scope of this paper. In addition, it is assumed that the rolling stock scheduling will not be influenced by the delays.
(A3) For simplicity, the metro line is assumed to have a straight and level track.
(A4) No stop-skipping is permitted in this paper.
(A5) The passenger flows are steady overtime.

### Graph representation

In the literature, Tomii [34] displayed train operation data in the form of a chromatic train diagram, because a timetable shows the train running states by providing the train index, position, speed, and time. Analogously, to consider train movement characteristics, the Train State Graph (TSG) proposed by Huang et al. [41], which consists of the train index, time, position, speed and train separation, and indicates the interactions between adjacent trains, is introduced here.

**Fig 2:**
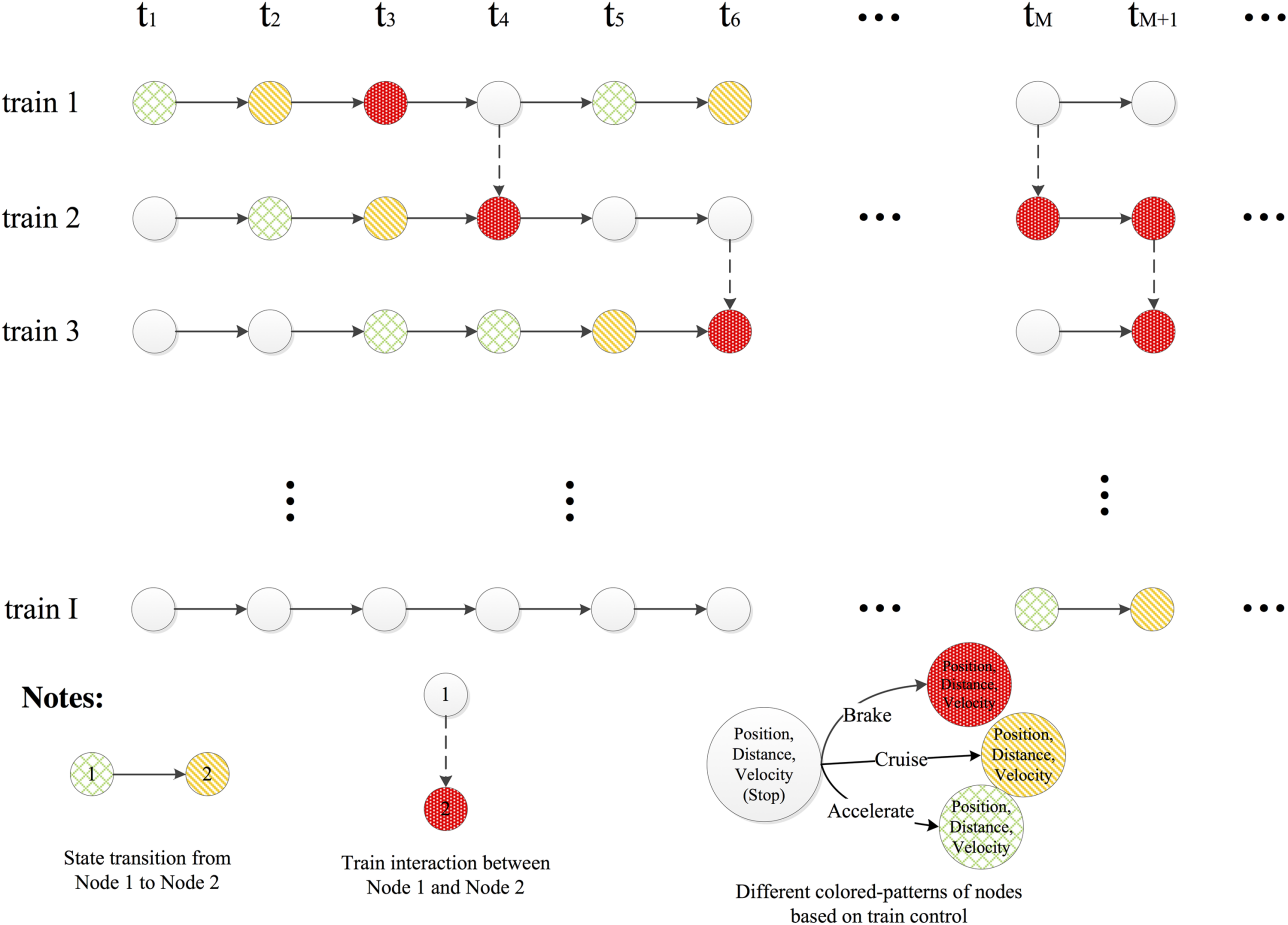
An example of train state graph.

As shown in Fig 2, TSG is a directed graph, with node set representing train states at different discrete events, and arc set representing train state transitions and train interactions. The discrete events in the graph consist of train arrivals and departures (macro-level), and train acceleration, cruising and deceleration (micro-level). The notations *t*_1_,…, *t*_*M*+1_,… represent the time stamps when the discrete events occur.

To analytically visualize the train movements in more details, the pattern-coding of the nodes is designed. Specifically, the white nodes without a pattern represent the complete stop of trains, the red nodes with dots represent the deceleration of trains, the green nodes with grids represent the acceleration of trains, and the yellow nodes with slashes represent the cruising of trains. If two nodes have different patterns, the subsequent node indicates the start of a new train state, and the end of the last state. For example, at *t*_4_, the node's pattern of train 2 turns from slashes to dots, showing that the train ends the state of cruising and starts deceleration. If two nodes have the same pattern, the subsequent node indicates that the train state remains.

The arc set can be divided into two subsets: the solid arc set and the dotted arc set. The solid arc set consists of the arcs linking two nodes of the same train, which represent train state transitions. For example, between time stamps *t*_2_ and *t*_3_, the arc for train 2 shows the change of control from acceleration to cruising, and the duration time for the acceleration is *t*_3_ − *t*_2_. The dotted arc set consists of the arcs linking two nodes of the adjacent trains, which consider train position, speed and train separation, and indicate the train interactions. Let *χ*_*s*_ ≥ 1, and *S*_*b*_ = *v*^2^ / (2 ⋅ *b*), where *v* is the instantaneous speed, and *b* is the maximum braking rate. Train interactions usually occur when the train separation between two trains is smaller than the required safety distance: *S*_*safe*_ = *S*_*M*_ + *χ*_*s*_ ⋅ *S*_*b*_.

TSG consists of all the essential events of the trains in the metro line. Given these events, the system state in any position at any time can be calculated. Besides, with analytic visualization, different types of the discrete events are displayed in corresponding patterns, and their relations are explicitly demonstrated with the arcs.

### Mathematical formulation

In this section, the energy consumption calculation model is introduced, after which we discuss how to account for the total delay. Then, the metro train rescheduling problem is formulated as a mixed integer programming model with time penalty and energy consumption.

#### Energy consumption calculation

In each section *k = 1*, *2*, *…*, *K-1*, we have the general train control equations:

$$u_{i}^{k}(t)=[F_{i}^{k}(t)-fr(v_{i}^{k}(t))]/m_{i}.$$ (1)

$$u_{i}^{k}(t)=\frac{dv_{i}^{k}(t)}{dt}.$$ (2)

$$v_{i}^{k}(t)=\frac{ds_{i}^{k}(t)}{dt}.$$ (3)

$$P_{i}^{k}(t)=F_{i}^{k}(t)\cdot v_{i}^{k}(t).$$ (4)

$$E_{i}^{k}(t)=\int_{t_{0}}^{t_{s}}P_{i}^{k}(t)dt.$$ (5)

Eqs (1), (2) and (3) represent train movements. Eqs (4) and (5) express the relations between the tractive effort and the energy consumption. *t*_0_ and *t*_*s*_ respectively represent the times when train *i* enters and leaves section *k*.

Combining Eqs (1), (2) and (5), Eq (5) is rewritten as follows:

$$\begin{array}{l} E_{i}^{k}(t)=\int_{t_{0}}^{t_{s}}[m_{i}\cdot u_{i}^{k}(t)+fr(v_{i}^{k}(t))]\cdot v_{i}^{k}(t)dt\\ =\int_{t_{0}}^{t_{s}}m_{i}\cdot vdv+\int_{t_{0}}^{t_{s}}fr(v_{i}^{k}(t))\cdot v_{i}^{k}(t)dt\\ =\int_{t_{0}}^{t_{s}}fr(v_{i}^{k}(t))\cdot v_{i}^{k}(t)dt. \end{array}$$ (6)

Eq (6) shows that with given section trip time stamps [*t*_0,_ *t*_*s*_], the speed profile determines the energy consumption. In other words, if an ideal speed profile for one section is given, the only factor that would influence the energy consumption is the trip time [*t*_0,_ *t*_*s*_], which is determined by the decision variables ($a_{i}^{k}$ and $d_{i}^{k}$).

Finally, the total energy of the affected trains is:

$$E_{total}=\sum_{i=1}^{I}\sum_{k=1}^{K-1}E_{i}^{k}.$$ (7)

#### Total delay

The delay $T_{delay}^{i}$ for train *i* can be calculated as the deviation between the original and actual arrival time of train *i* at the terminal station *K*_*s*_:

$$T_{delay}^{i}=a_{i}^{K_{s}}-A_{i}^{K_{s}};\forall i\in I.$$ (8)

Since the trains without delay effects will arrive at their destination stations on time, the total delay $T_{delay}^{total}$ in the metro line is the sum of the delays of all affected trains:

$$T_{delay}^{total}=\sum_{i=1}^{I}T_{delay}^{i}.$$ (9)

In the objective function, passenger demands are not considered. However, in actual peak hours, the delay will increase the number of passengers arriving and waiting at the stations. To guarantee the safety of the passengers, the capacity of the platforms should be considered. Let $Q_{i}^{k}=(d_{i}^{k}-d_{i-1}^{k})\cdot \lambda^{k}$, the total number of passengers on the platform of station *k* when train *i* arrives can be calculated as:

$$N_{i}^{k}=N_{i-1}^{k}+\alpha_{i-1}^{k}-\beta_{i-1}^{k}+Q_{i}^{k};\forall 2\leq i\leq I,1\leq k\leq K.$$ (10)

In each station *k*, the platform capacity should not be exceeded:

$$N_{i}^{k}\leq Cap^{k}.$$ (11)

No train capacity constraint is assumed in this paper. That is, each train is assumed to be able to receive all the boarding passengers.

#### Optimization model

The delays would increase energy consumption by forcing the succeeding trains to frequently decelerate and re-accelerate, and increase the delays in the succeeding trains due to the propagation effect. Thus, the optimization model aims to minimize a weighted cost of the energy consumption and travel delay under delay scenarios, by optimizing the affected section trip times. It should be noted that according to Assumption (A5), the variability of passenger flows is not considered, and thus the dwell times of the affected trains are the same as in the original timetable. In this regard, optimizing the affected section trip times is equivalent to optimizing the decision variables in the model.

As mentioned above, there are trade-offs between the energy consumption and the total travel delay. Considering these tradeoffs, we use two weighting factors *w*_*e*_ and *w*_*t*_ for the relative importance of these indicators. Furthermore, to generalize the cost of both energy and time, we define the unit cost of energy consumption as *c*_*e*_ (RMB yuan/Kwh), and the unit cost of delay (regarded as the penalty cost) as *c*_*t*_ (RMB yuan per hour). The optimization model is formulated as below.

Minimize

$$C_{total}=w_{e}\cdot c_{e}\cdot E_{total}+w_{t}\cdot c_{t}\cdot T_{delay}^{total}.$$ (12)

Subject to

$$0\leq a_{i}^{k}-A_{i}^{k}\leq \eta^{k};\forall 1\leq i\leq I,1\leq k\leq K.$$ (13)

$$0\leq d_{i}^{k}-D_{i}^{k}\leq \eta^{k};\forall 1\leq i\leq I,1\leq k\leq K.$$ (14)

$$\underline{\omega^{k}}\leq d_{i}^{k}-a_{i}^{k}\leq \bar{\omega^{k}};\forall 1\leq i\leq I,1\leq k\leq K.$$ (15)

$$\underline{t^{k}}\leq a_{i}^{k+1}-d_{i}^{k}\leq \bar{t^{k}};\forall 1\leq i\leq I,1\leq k\leq K-1.$$ (16)

$$\underline{\{\begin{array}{c} d_{i}^{k}-d_{i-1}^{k}\geq h_{\text{min}}\\ a_{i}^{k}-a_{i-1}^{k}\geq h_{\text{min}} \end{array}};\forall 2\leq i\leq I,1\leq k\leq K.$$ (17)

$$N_{i}^{k}\leq Cap^{k};\forall 1\leq k\leq K.$$ (18)

$$a_{i}^{k},d_{i}^{k}\in {\mathbb{Z}}^{+};\forall 1\leq i\leq I,1\leq k\leq K.$$ (19)

Note that since the unit of energy (J) is different from the corresponding unit in *c*_*e*_ (Kwh), as well as the unit of delay (s) and the corresponding unit in *c*_*t*_ (hour), the unit conversion (1 hour = 3600 s, and 1 Kwh = 3.6·10^6^ J) should be considered in Eq (12). Constraints (13) and (14) restrict that the deviation of arrival and departure times of the affected trains between the optimized and the original timetables should be within the threshold value. Constraints (15) and (16) limit the dwell and travel times of the affected trains. Constraint (17) indicates that the headway between adjacent trains must not violate the minimum value, to ensure the trains travel safely. Constraint (18) guarantees that the number of passengers on the platform will not exceed its capacity. Constraint (19) requires that the arrival and departure times of trains should be positive integers, with units of seconds.

Since many timetables have buffer times to absorb small delays, usually a primary delay could only affect several subsequent sections. In addition, as aforementioned, the aim of train rescheduling is to change the original timetable as little as possible, while minimizing the profit loss (i.e., the weighted cost in this paper). Therefore, to reschedule all trains in all their remaining sections is neither economic nor necessary. In this work, we develop a novel method to accurately identify the affected trains in affected sections, thereby considerably reducing the scale of decision variables.

## Solution approach

To introduce the algorithms with clarity, two definitions should first be explained: (1) Flow-on delay is defined as a delay observed in the succeeding train at a station or in a section, which is caused by a primary delay but has no direct relation with its preceding train; (2) Flow-on effect is equivalent to a train interaction. It is defined as a direct effect in the succeeding train that is caused by a delay in its preceding train, and it is usually seen as an unexpected deceleration in the train running states. Generally, a flow-on effect occurs in the succeeding train when it enters the moving block of its preceding train, i.e., the distance between these two trains is smaller than the safe distance *S*_*safe*_. The solution approach focuses on the flow-on effects, because they reveal the relations among trains with more information, such as the time stamp, train speed, position and train control.

According to Caprara et al. [42], the train optimization problem is an NP-hard problem. Traditionally, to find a good solution, heuristic algorithms are adopted, such as genetic, simulated annealing, and particle swarm algorithms. In this paper, a two-stage solution approach is proposed. The first stage is a primary delay identification algorithm, which will identify the primary and flow-on delays, as well as their flow-on effects, thereby forming critical paths for each primary delay. The second stage is a hybrid-genetic algorithm, which is designed to generate near-optimal solutions for the MIP model. Based on the identification results, the hybrid-GA is expected to have better computation performance and more practical results.

### Primary delay identification algorithm

The primary delay identification algorithm (PDIA) is essentially a critical path algorithm which considers the interrelations among trains, and explores the possibilities of parallel computing in larger testbeds. As shown in TSG, the train interactions are denoted as the dotted arcs, the delays are expressed as a series of continuous white nodes (train stops and waits in a section or is delayed at a station), and the flow-on effects are denoted as the frequent dotted nodes. Each flow-on effect forces the succeeding train to decelerate (dotted node linked with a dotted arc), thereby causing the flow-on delay. In this way, a critical path can be formed by tracing back a sequence of dotted arcs and white/dotted nodes which represent flow-on delays and effects until a primary delay is identified. Thus, the PDIA aims to identify different types of delays (primary and secondary) and train interactions (caused by those delays) from train running data (represented by the TSG), as well as classify these results by different primary delays (each primary delay corresponds to a critical path).

Define four sets: *U*_*P*_, *U*_*F*_, *U*_*T*_ and *U*_*S*_, which express the primary delay, the flow-on effects and delays, the time stamps when the flow-on effects and delays occur, and the affected stations/sections, respectively. Let ‖*U*‖ be the number of elements in set *U*. These sets satisfy the condition:‖*U*_*P*_‖ = ‖*U*_*F*_‖ = ‖*U*_*T*_‖ = ‖*U*_*S*_‖. Now, each train with primary delay {*U*_*P*_}_*x*_, ∀1 ≤ *x* ≤‖*U*_*P*_‖ corresponds to a subset of trains with flow-on effects and delays in {*U*_*F*_}_*x*_, a subset of time stamps in {*U*_*T*_}_*x*_, and a subset of section index in {*U*_*S*_}_*x*_. For example, given the first elements in all the four sets as: {*U*_*P*_}_1_ = (1, 5), {*U*_*F*_}_1_ = (2, 2, 3, 3)^*T*^, {*U*_*T*_}_1_ = (120, 180, 200, 260)^*T*^ and {*U*_*S*_}_1_ = (4, 5, 4, 5)^*T*^. We can conclude the following relations: Train 1 has the primary delay at station 5, which generates the flow-on effect of train 2 at time 120s in section 4; The flow-on delay of train 2 starts at time 180s at station 5, which generates the flow-on effect of train 3 at time 200s in section 4; The flow-on delay of train 3 starts at time 260s at station 5. Therefore, we have ‖{*U*_*F*_}_*x*_‖ = ‖{*U*_*T*_}_*x*_‖ = ‖{*U*_*S*_}_*x*_‖, ∀1 ≤ *x* ≤‖*U*_*P*_‖. In this way, the effects and delays are classified, linked to one another, and represented by critical paths. Each critical path starts from a given point, tracing along the flow-on effects and delays, and ends in a primary delay.

The aim of PDIA is to identify different types of delays in the system, and then form the critical paths. It is divided into two sub-algorithms: (1) the Quasi-MapReduce Search (QMS) algorithm aiming to identify the primary and flow-on delays and their flow-on effects, and (2) the Synchronized Iterative Path Searching (SIPS) algorithm aiming to form the critical paths.

#### QMS algorithm

The QMS algorithm is performed only once for each data set. In the Map function, the algorithm searches the whole data set, and selects the records by Rule 1. In the Reduce function, it adds Map results into the output set *D*. QMS is designed as follows:

Data preparation: Arrange the train operation data. For each train operation record, set the key as train index (*i*), and the values as: time (*t*), position (*s*), instantaneous speed (*v*), adjacent train distance (*dis*), current section (*k*), and control strategy (*o*). The data arrangement can be completed by existing algorithms (Idris et al. [43]).

Rule 1: If the record satisfies Rule 1, it must meet the following requirements: (1) Its value of control strategy *o* represents a deceleration strategy (*o = 2*); (2) Its value of speed *v* must not violate the minimum speed, which is denoted as *v*_min_; (3) Its value of position *s* and adjacent distance *dis* must indicate that this train and its preceding train move in the same section (*s* + *dis* ≤ *s*_*k*+1_, where *s*_*k*+1_ is the position of next station).

Algorithm 1 Map Function

For Each train operation record do

 If the record satisfies Rule 1, then

  Find its “pair record”^**1**^ (if the pair record does not exist, return 0)

   If The pair record is not 0, then

    If *dis* < *χ*_*s*_ ⋅ *S*_*b*_ + *S*_*M*_, then

     Emit the record and its pair record, with train index as the key, and its

     values are *t*, *s*, *v*, *dis*, *k* and *o*.

    End if

   End if

  End if

 End for

*Remark 1*: The “pair record” refers to such a train operation record that its key is the preceding train index, with the value of time *t*, and the value of position *s+dis*.

It should be noticed that in the Map function, most noise data will be filtered out by Rule 1, because the noise data that represent unexpected train controls and other irrelevant events are expected to have the values of *v* < *v*_min_ or *s* + *dis* > *s*_*k*+1_.

Reduce function: Since all the computation was performed in the Map phase, the identity reducer (Lin and Dyer [44]) is used here to ensure that the output data set *D* is sorted by key (train index *i*).

#### SIPS algorithm

The SIPS algorithm is an iterative algorithm, which traverses the data set *D* from QMS algorithm, classifies the primary and flow-on delays in the QMS result, and connects data to form the critical paths. The set *U*_0_ = *U*_*P*_ ∪ *U*_*F*_ ∪ *U*_*T*_ ∪ *U*_*S*_ is designed here to store the elements that constitute the critical paths. The Synchronized Iterative Path Search algorithm is designed as follows:

Algorithm 2 Synchronized Iterative Path Search Algorithm

Step 1: Collect the output data *D* from QMS. Initialize *U*_0_ = ∅. Set *count*_*data*_ = ‖*D*‖, and *x = 1*.

Step 2: For the record with index *count*_*data*_ in *D*: If *o = 2*, go to Step 2.1; Otherwise, go to Step 2.2.

Step 2.1: The record with index *count*_*data*_ represents the flow-on effect. Add its key (train index *i*) into {*U*_*F*_}_*x*_, its value of time *t* into {*U*_*T*_}_*x*_, and its value of section *k* into {*U*_*S*_}_*x*_. Based on the QMS result, set *count*_*data*_ = *z*_*p*_, where *z*_*p*_ is the record index in *D* whose value of position is the same as the current record, and return to Step 2.

Step 2.2: The record with index *count*_*data*_ represents the train delay. Perform “Data reverie”^**2**^. If the data reverie returns a record, go to Step 2.2.1; Otherwise, go to Step 2.2.2.

Step 2.2.1: The record with index *count*_*data*_ represents the flow-on delay. Add its key *i* into {*U*_*F*_}_*x*_, and add its values of *t* and *k* into {*U*_*T*_}_*x*_ and {*U*_*S*_}_*x*_, respectively. Set *count*_*data*_ = *z*_*new*_, and return to Step 2.

Step 2.2.2: The record with index *count*_*data*_ represents the primary delay. Add its key *i* and station index *k* into {*U*_*P*_}_*x*_, and set *x = x+1*. Go to Step 2.3.

Step 2.3: Update *D*: in the set *D*, delete the elements which have been stored in the set *U*_0_. Set *count*_*data*_ = ‖*D*‖. If *count*_*data*_ = 0, go to Step 3; Otherwise, return to Step 2.

Step 3: Output the results: *U*_*P*_, *U*_*F*_, *U*_*T*_ and *U*_*S*_.

*Remark 2*: The process of “data reverie” aims to find the relations among records that share the same key, and consists of two steps: (1) Given a key *i* of a record whose value of position is *s* = *s*_*r*_, traverse the other records of train *i*, and find the record with position value *s* = *s*_*new*_, where *s*_*new*_ should satisfy the following condition: 0 < *s*_*r*_ − *s*_*new*_ ≤ *s*_*r*_ − *s*_*key*_, ∀*s*_*key*_ ∈ *S*_*key*_, and *s*_*key*_ is the set of position values that satisfy *s* < *s*_*r*_. (2) If *s*_*new*_ represents that the train is at a station, this delay is not caused by the flow-on effects in the former section. Thus, it is a primary delay, and return null; Otherwise, return the record with index *z*_*new*_ whose position value is *s* = *s*_*new*_.

Note that the correspondence of each element in the *U*_*P*_ and *U*_*F*_ demonstrates the start, middle and end points of one path; *U*_*T*_ indicates the time stamps of the flow-on effects and delays; *U*_*S*_ shows the affected stations/sections. In this regard, the critical paths in the metro system can be determined by the outputs of SIPS algorithm. Finally, the Main Algorithm is designed as follows:

Algorithm 3 Primary Delay Identification Algorithm

Step 1: Initialization. Set *U*_*P*_ = ∅, *U*_*F*_ = ∅, *U*_*T*_ = ∅, and *U*_*S*_ = ∅. Input the original train operation records.

Step 2: Perform QMS and obtain the set of results *D*.

Step 3: Based on the data set *D*, perform SIPS.

Step 4: Output *U*_*P*_, *U*_*F*_, *U*_*T*_ and *U*_*S*_.

### Hybrid genetic algorithm

A genetic algorithm is proposed here for optimizing the solution. Based on the PDIA results, the indexes of affected trains, sections and stations are determined. Thus, the number of the decision variables decreases, by considering only the trip times of affected trains in affected sections, instead of all their remaining journeys. In the genetic algorithm, the smaller input scale leads to not only smaller chromosome length, but also simpler calculation of the fitness value, selection, crossover, and mutation process, thereby improving the algorithm efficiency.

Since the GA is a well-known algorithm, we only show the modifications of the generic GA here, which improves its effectiveness and efficiency. The complete program for a generic GA can be referred in Xu et al. [45].

#### Modification in the “Initialization” process

**Fig 3:**
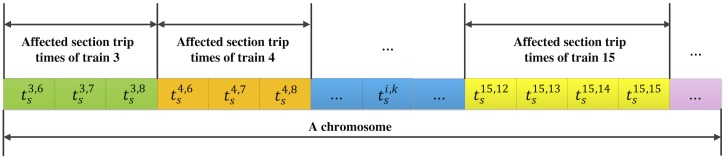
Representation of a chromosome.

In the hybrid-GA, the generation process of feasible chromosomes in the generic GA is modified. Assume that there are *size*_*pop*_ feasible chromosomes in one generation. We first denote a gene in one chromosome as $t_{s}^{i,k}$, which represents the trip time for an affected train *i* in an affected section *k*. A chromosome then consists of all those trip times that needs to be optimized, which represents a complete solution to the optimization model, as shown in Fig 3. To provide good chromosomes, we make the following modifications: (1) For each $t_{s}^{i,k}$, find the minimum value $\underline{t_{s}^{i,k}}$ and the maximum value $\bar{t_{s}^{i,k}}$, based on the simulation result (which determines $\underline{t_{s}^{i,k}}$) and the model constraint (which determines $\bar{t_{s}^{i,k}}$); (2) Create the alternative gene value set Γ, whose elements can be calculated as:$\Gamma_{j}=\underline{t_{s}^{i,k}}+(\bar{t_{s}^{i,k}}-\underline{t_{s}^{i,k}})\cdot [(j-1)/(size_{pop}-1)],\forall j=1,2,\ldots ,size_{pop}$; (3) Generate randomly Ψ ∈ (0,1), calculate *x* = ⌈*size*_*pop*_ ⋅ Ψ⌉, where ⌈*x*⌉ returns the closest integer which is larger than *x*; (4) Set the gene value as Γ_*x*_. The other processes of the initialization are the same as in the generic GA, including the feasibility testing process for each chromosome (the model constraints (13)–(19) are all considered), and the iterating process for generating different chromosomes until the number reaches *size*_*pop*_.

#### Calculating the fitness value

Since the gene $t_{s}^{i,k}$ is equivalent to the decision variables $a_{i}^{k}$ and $d_{i}^{k}$, i.e., $a_{i}^{k+1}-d_{i}^{k}=t_{s}^{i,k}$(the maximum section index is*K-1*), the fitness value of one chromosome can be obtained by calculating the objective function with respect to $t_{s}^{i,k}$. Then, to distinguish a good chromosome from those bad or infeasible chromosomes, we define a simple penalty term *ξ*. Specifically, if the chromosome is infeasible or the number of train influences detected by the QMS algorithm increases, we have *ξ = +*∞; Otherwise, we have *ξ* = 0. It should be noted that since the results of QMS algorithm suffice to determine whether the solution is improved, to reduce computation time, the SIPS is not performed here.

We add *ξ* to the objective function, and based on Eq (12), we have the fitness value function used in the hybrid GA:

$$C_{affected}=w_{e}\cdot c_{e}\cdot E_{affected}+w_{t}\cdot c_{t}\cdot T_{delay}^{affected}+\xi .$$ (20)

where *C*_*affected*_, *E*_*affected*_ and $T_{delay}^{affected}$ are the objective functions corresponding to the identification results from PDIA. This design integrates the genetic algorithm with our QMS algorithm, and thus the new GA is a hybrid one. Finally, based on Eq (20), we can calculate the fitness value corresponding to each chromosome.

#### General scheme of Hybrid-GA

The processes of ranking, selection, crossover and mutation are mainly the same as in the generic GA, and thus are not described here. Finally, the general scheme of Hybrid-GA is described as follows.

Algorithm 4 Hybrid Genetic Algorithm

Step 1: Set probability for crossover *P*_*c*_, probability for mutation *P*_*m*_, total population of one generation *size*_*pop*_, maximum generation number max_*gen*_. Set *count* = 1.

Step 2: Initialize the first generation with *size*_*pop*_ chromosomes.

Step 3: Calculate the objective function, and sort the chromosomes from small to large. Update the best fitness value, the best individual, and the best generation.

Step 4: Breed the new generation with selection, crossover, and mutation operations. *count* = *count* + 1.

Step 5: If *count* = max_*gen*_, go to Step 6; Otherwise, return to Step 3.

Step 6: Output the best fitness value, the best individual, and the best generation. Stop the algorithm.

## Numerical case studies

To show the effectiveness and efficiency of the proposed solution approach, the two steps are separately implemented based on the actual Beijing Subway Line 4 (BSL4). In the first step, we demonstrate how the primary delay identification algorithm is used to classify the delays and form the critical paths. In the second step, we present the optimization solutions from the Hybrid-GA (HGA), with smaller input scale based on the PDIA results. The computational advantage of HGA is tested with a comparison between HGA and the generic GA (GGA) that takes all remaining sections of affected trains as input.

BSL4 is a busy line with 35 stations, connecting the central business districts, the advanced technological zones and the suburban residential areas. More infrastructure information and line representation can be referred in Xu et al. [45] or the website: https://en.wikipedia.org/wiki/Line_4,_Beijing_Subway. Here we specifically discuss the train rescheduling in one hour (3600s) during the peak time, in one direction from AHQB to TGY. In the original plan, the trains are uniformly distributed within one hour, which is the case in the real-world. Table 2 lists parameters of train running. The computational results are obtained on a personal computer with 2GHz Intel Corei7 CPU and 8GB memory.

**Table 2:** Parameters of trains in the BSL4.

Parameter	Unit	Value
Train mass (*m*_*i*_)	Kg	2×10^5^, ∀1 ≤ *i* ≤ *I*.
Constant acceleration rate	m/s^2^	1.2
Constant deceleration/braking rate	m/s^2^	1
Train resistance function (*fr*(*v*))	m/s^2^	1.36 ⋅ 10^−4^ ⋅ *v*^2^ + 1.45 ⋅ 10^−2^ ⋅ *v* + 0.8
Minimum headway (*h*_min_)	s	100
Safety margin (*S*_*M*_)	m	270
Threshold deviation value (*η*^*k*^)	s	130, ∀1 ≤ *k* ≤ *K*.
Total number of trains	-	36

### PDIA performance evaluation

Let (*i*, *k*) be one given delay (in seconds) for train *i* at station *k*. Consider the following three cases: (1) Case a: Based on the characteristics of passenger distribution, HDHZ, XZM and BJSRS are the busiest stations. Assume the delays occur at these stations: (5,20) = 135s; (7,17) = 152s; (10,12) = 135s; (13,11) = 139s; (16,6) = 127s. To further evaluate the algorithm performance, and test if it can identify the running delays (e.g. train costs more time in section than planned), Case b and Case c are introduced. (2) Case b: The basic case, with given delays as: (4,7) = 130s; (5,12) = 130s; (6,17) = 138s; (7,21) = 136s; (8,13) = 129s; (9,27) = 130s. (3) Case c: The comparison case, with given delays as: (4,7) = 130s; (5,12) = 130s; (6,17) = 138s; (7,21) = 136s; (8,13) = 129s; (9,27) = 130s. The section trip time for train 6 in section 17 in Case c is delayed by 120s.

Considering the given delays, a simulation method is used to complete the original train timetables in different delay scenarios, which is referred in Xu et al. [23]. The simulation results show that the trains travel densely with small separations. Thus, a small delay may cause severe disturbances in the system. The PDIA is then performed in these three cases, to find train interaction relations among the data. Table 3 lists the main numerical results of PDIA, where “TNI” represents the total number of the influences caused by the primary delays, and “Accuracy” represents the ratio between the number of primary delays identified by PDIA and the given delays. Table 4 lists some critical path elements in Case a.

**Table 3:** Main numerical results of PDIA.

	Simulation time (s)	CPU time (s)	TNI	Accuracy
Case a	38.524	1.346	3226	100%
Case b	43.620	1.587	8723	100%
Case c	46.496	1.704	10966	100%

**Table 4:** Examples of critical path elements in Case a.

*U*_*P*_	*U*_*F*_	*U*_*T*_	*U*_*S*_
(5, 20)	(6, 6, 7, 7)	(3509, 3547, 3577, 3724)	(19, 20, 19, 20)
(7, 17)	(8, 8, 9, 9)	(3269, 3305, 3345, 3381)	(16, 17, 16, 17)
(10, 12)	(11, 11, 12, 12)	(2822, 2860, 2939, 2979)	(11, 12, 11, 12)
(13, 11)	(14, 14, 15, 15)	(2898, 2934, 2967, 3008)	(10, 11, 10, 11)
(16, 6)	(17, 17, 18, 18)	(2416, 2448, 2486, 2521)	(5, 6, 5, 6)

Table 3 indicates that PDIA is effective and efficient, identifying the primary delays with 100% accuracy very fast. As expected, in comparing Cases a, b and c, we find that more occurrences of primary delays lead to a higher TNI value (Case c>b>a), and the substantial increase of TNI from Case b to Case a shows that the effects of primary delays will propagate and accumulate. Therefore, the primary delays and their flow-on effects should be dealt with as soon as possible. In addition, Table 4 highlights part of the affected train index, time stamp and station/section index. Each row in Table 4 contains the information to form a critical path that corresponds to an identified primary delay. For instance, the primary delay of train 5 at station 20 would affect the ideal travels of trains 6, 7, 8 and 9 (the information of trains 8 and 9 is not listed in Table 4, but they are identified in the complete results). The affected time stamps for the flow-on effect of train 6 in section 19 is 3509s, which causes the flow-on delay of train 6 starting at 3565s at station 20. Therefore, a critical path is formed, which starts from the last flow-on delay (train 9 in section 21), traces back through other flow-on delays and effects, until the primary delay (5, 20) is reached. These results will minimize the size of input of Hybrid-GA. Moreover, the TNI of the timetable without the given delays in Case a is 481, which proves that to identify the primary delays is an essential way to decrease the train interactions, thereby reducing the unnecessarily frequent acceleration and deceleration controls.

**Fig 4:**
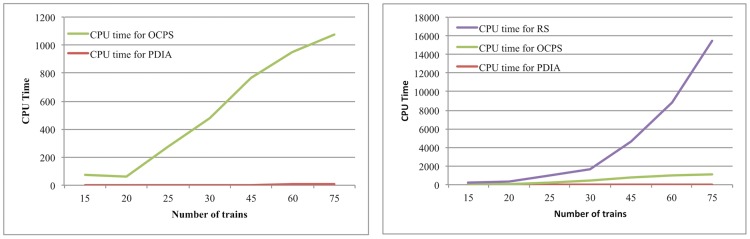
CPU time (s) for different methods.

Fig 4 shows the comparison of computation time for different primary delay identifying methods in the larger testbeds: (1) Original critical path search algorithm (OCPS) without parallel computing (Huang et al. [41]), which is designed for searching one path at a time; (2) Repeated-simulation Searching algorithm (RS), which aims to identify the primary delays by comparing simulation results; and (3) PDIA. It is obvious that PDIA greatly reduces the computation time needed for identifying the primary delays. Thus, the effectiveness and efficiency of PDIA are demonstrated through this example.

### Hybrid-GA performance evaluation

With the input from the PDIA results, the Hybrid-GA is adopted to reschedule the train timetable. It should be noted that the rescheduling process is adopted after the latest delay has occurred, and thus some of the section trip times cannot be optimized. For example, the delay (16,6) = 127s impacts the succeeding train motions in sections 5 and 6, but all trains have passed section 6 when the latest delay occurs. In this regard, the input of Hybrid-GA does not contain the variables of trip times in section 5 or 6. This is consistent with practical experiences, where dispatchers could only make the best decision for future operations. That is, the optimized variables in the Hybrid-GA include all trip times of affected trains in the affected sections after the latest delay.

**Fig 5:**
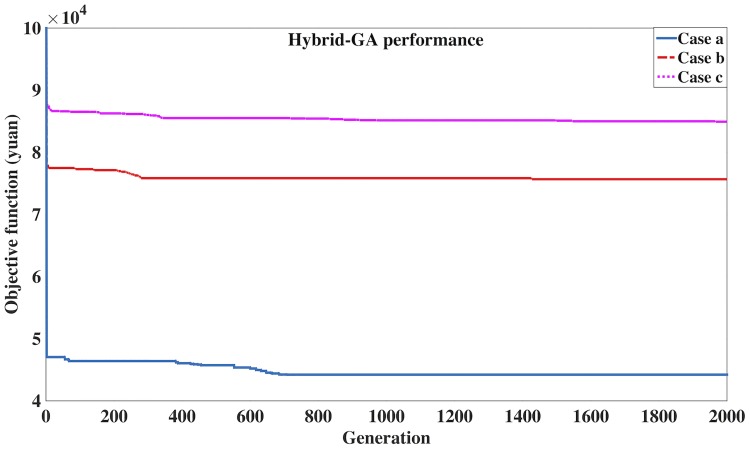
Fitness value in each generation of Hybrid-GA (Cases a, b and c).

The algorithm parameters are: (1) Maximum generation: 2000; (2) Population size of chromosomes in one generation: 20; (3) Crossover probability: 0.7; (4) Mutation probability: 0.3; (5) Weightings of energy and travel time: *w*_*e*_ = 1, *w*_*t*_ = 1; (6) Cost of energy (yuan/Kwh): 0.79, and cost of time (yuan per person per hour): 20. In addition, the average number of on-board passengers per train is 1500, and the average weight per passenger is 75Kg. The optimized solution in Case a is shown in Table 5, and the computational performances of Hybrid-GA in Cases a, b and c are shown in Table 6, where the generic GA performances are shown for comparisons. Fig 5 shows the best fitness in each generation of the three cases in Hybrid-GA.

**Table 5:** Solution of Hybrid-GA in Case a.

	**6**	**7**	**8**	**9**	**10**	**11**	**12**	**13**	**14**	**15**	**16**	**17**	**18**	**19**	**20**
13	-	-	-	-	-	-	-	-	-	-	-	-	-	158s	160s
14	^-^	-	-	-	-	-	-	-	-	-	-	152s	146s	145s	151s
15	-	-	-	-	-	-	-	-	-	-	129s	128s	127s	126s	-
16	-	-	-	-	-	-	-	105s	116s	136s	112s	118s	-	-	-
17	-	-	-	-	-	-	-	117s	112s	129s	111s	-	-	-	-
18	-	-	-	-	195s	188s	185s	185s	178s	-	-	-	-	-	-
19	244s	146s	151s	158s	156s	153s	152s	158s	-	-	-	-	-	-	-
20	255s	242s	208s	220s	222s	247s	245s	-	-	-	-	-	-	-	-
21	191s	212s	190s	195s	175s	-	-	-	-	-	-	-	-	-	-

**Table 6:** Computational results for HGA and GGA.

	HGA time (s)	GGA time (s)	Original cost (yuan)	After HGA (yuan)	Rate (%)	After GGA (yuan)	Rate (%)
Case *a*_1_	251.9	889.7	52284	**44223**	**15.42**	49657	5.02
Case *a*_2_	226.6	889.7	52284	**44385**	**15.11**	49657	5.02
Case b	435.8	1752.9	87608	**75676**	**13.62**	80797	7.77
Case c	453.9	1932.6	99867	**84939**	**14.95**	89178	10.70

Fig 5 indicates that Hybrid-GA converges in all the three cases, and quickly reaches a near-optimal solution. It iteratively searches for a better solution in each generation. The best individuals are found at the 709th, 600th, 1201st generation, in Cases a, b and c, respectively. Table 5 demonstrates that number of the decision variables is significantly reduced. Specifically, the rows represent section indexes and the columns represent train indexes. For example, the cell corresponding to the row with index “19” and the column with index “13” represents the optimized trip time of train 19 in section 13. The section trip times in the optimization solution are restricted by the upper bound in the model constraints, and the lower bound in the PDIA results. Thus, the train interactions are mostly reduced, with longer trip times for the affected trains in affected sections. Moreover, due to the time penalty in the objective function and the train headway restrictions in the model constraints, the schedule adherence requirements of the optimized train timetable are satisfied. The values of decision variables of the optimization model can be calculated based on the Hybrid-GA solution.

Table 6 shows the effectiveness and computation advantage of the proposed Hybrid-GA. Cases *a*_1_ and *a*_2_ are two independent results obtained by running the genetic algorithms twice, both of which are based on the delay scenario of Case a. The total cost is significantly reduced after applying Hybrid-GA. From the optimization results of Cases *a*_1_ and *a*_2_, similar computational performances are obtained. Thus, it is verified that the HGA can reach near-optimal solutions. In addition, with the smaller input (44 in HGA compared to 278 in GGA), the computation time of Hybrid-GA is greatly reduced (e.g., Case *a*_2_: 226.6s compared to 889.7s in GGA). The cost reduction rates of HGA are also greater than GGA, which could be further explained. GGA accounts for all the trip times of the affected trains in remaining sections, while HGA assumes the trip times in those unaffected sections are ideal. Since the simulation method would consider the short headway and generate some train interactions that are not related to the primary delays (the same reason why the timetable without the primary delays still has the TNI value as 481), the time penalty in the objective function for GGA is larger than for HGA. As train rescheduling aims to minimize changes of the original timetable, the HGA solution is preferable and more practical for rescheduling. In each case, the computation time of HGA is less than 8min. Note that the experiments are implemented in a large-scale case: in a busy hour of a 35-station long metro line, with the dense 100s headway between adjacent trains. That is, the proposed algorithm is applicable for real-time train rescheduling.

Above all, the proposed Hybrid-GA is demonstrated to be both effective and efficient, and outperforms the generic GA.

### Sensitivity analyses

To further demonstrate the performance of the algorithm and find more interesting conclusions, sensitivity analyses are conducted, by adopting different parameters for Beijing Subway Line 4.

(1) In this experiment, the influence of different weighting coefficients in the objective function is tested. Based on Case a, different pairs of weighting coefficients, i.e., (*w*_*e*_, *w*_*t*_), are applied in the objective function. The rescheduling results are shown in Table 7.

**Table 7:** HGA solutions with respect to different weighting coefficients in Case a.

(*w*_*e*_, *w*_*t*_)	(0, 2)	(0.5, 1.5)	(0.8, 1.2)	(1.2, 0.8)	(1.5, 0.5)	(2, 0)
HGA solution (yuan)	39950	42725	44106	45143	46380	46626
Delayed time (s)	2623	2535	2582	2562	2609	3435
Energy (Kwh)	30157	30250	30202	30202	30217	29692

The weighting coefficients indicate the emphasis of operation. With *w*_*e*_ > *w*_*t*_, the energy consumption is more important in the optimization objective, and vice versa. Two extreme cases are shown in Table 7, i.e. *w*_*e*_ = 2, *w*_*t*_ = 0 and *w*_*e*_ = 0, *w*_*t*_ = 2. In these two cases, only one factor is considered in the objective function. The results show that when delay is considered in the objective function, the weighting coefficients do not significantly influence the optimal performance. Therefore, the time penalty is important for the timetable adherence. In addition, with more weighting on delays, the total economic cost is lower. The reason might be that in the experiments, the unit cost of time (20 yuan per person per hour) is a little higher than the average income in China (15 yuan per person per hour), while the energy cost is realistic. It can be inferred that with the increase of per capita income, punctuality will become a more important factor in future operations.

**Fig 6:**
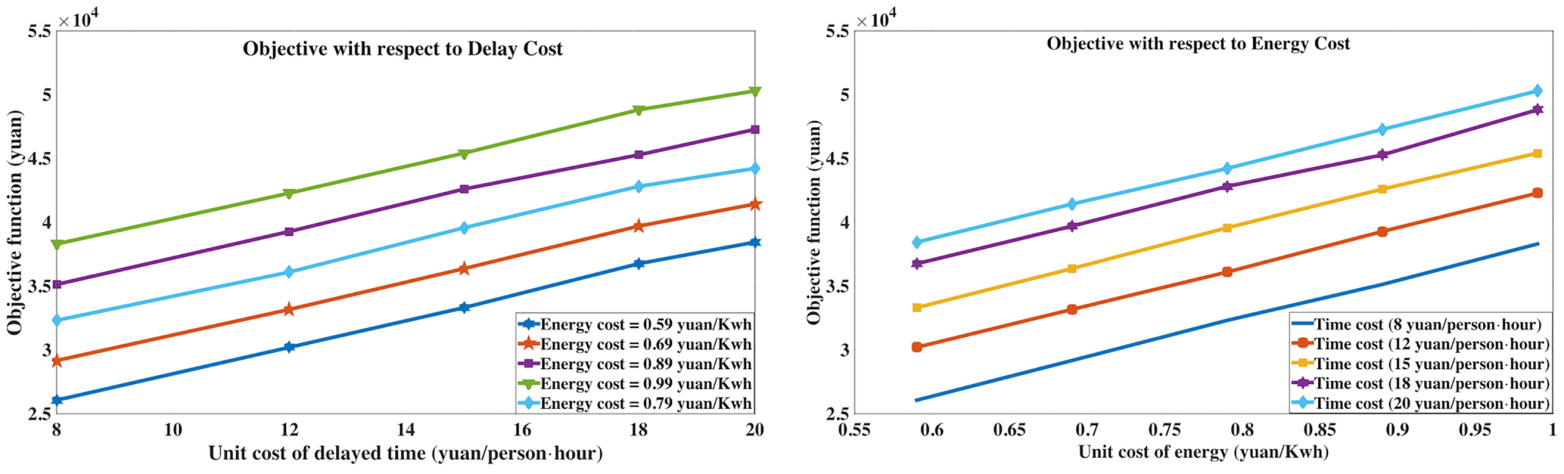
The influence of unit cost for optimal objective.

(2) In the second experiment, the sensitivity of the solution with respect to the unit costs of energy and travel delay is analyzed. Based on Case a, different sub-optimal solutions are generated for different unit costs (*c*_*e*_, *c*_*t*_) (*w*_*e*_ = 1, *w*_*t*_ = 1), as shown in Fig 6. The unit costs significantly influence the optimized objective function values, and the relations between the factor and the objective are both near-linearly and monotonically increasing in the figures, as the objective function of the proposed model indicates. Therefore, the model and the solution approach is tested to be reasonable and consistent.

## Conclusion

Aiming to reschedule online the affected trains in delay scenarios, this study first simplifies the optimization problem with the train state graph, and formulates the problem with a mixed integer programming model. A two-stage solution approach is then designed to obtain the optimization solution for the proposed model. The primary delay identification algorithm in the first stage is a critical path algorithm, which has a parallel computing design based on the graph representation. The hybrid-GA in the second stage adopts the PDIA results, so that the input size is significantly reduced, and the trains will be rescheduled as slightly as possible. The case studies based on Beijing Subway Line 4 demonstrate the efficiency and effectiveness of the proposed solution approach. PDIA results show the accuracy and efficiency of the proposed algorithm, which is tested to have computational advantages compared to the other critical path algorithms. The Hybrid-GA is demonstrated to be much faster than the generic GA that takes all remaining sections of affected trains as input. The solution of HGA is tested to reach near-optimality, with more than 10% reduction of total cost in each case, which is more practical than the generic GA. Finally, in the sensitivity analyses, the model is shown to be consistent, and the discussions on the parameters imply that the proposed solution approach can be developed as a decision-making support tool for operators.

Future studies will focus on the extensions and modifications of the proposed solution approach. One possible extension is to identify more types of the disturbances in the real world, such as infrastructure failures, signal errors, and unexpected stops of trains due to inappropriate driver’s behaviors. The patterns of train operation data and the relations among them are very much alike to those in the primary delay scenario. Other extensions and modifications may be considered for faster and better computational results.

## Supporting information

S1 Text Checklist and descriptions of supporting files. (DOCX)

S1 Dataset Complete simulation results of Case a in the numerical case study. (ZIP)

S2 Dataset Complete simulation results of Case b in the numerical case study. (ZIP)

S3 Dataset Complete simulation results of Case c in the numerical case study. (ZIP)

S4 Dataset Supplementary results for PDIA and Hybrid-GA in the numerical case study. (ZIP)
