# Supplementary material for: Real-time energy-saving metro train rescheduling with primary delay identification
Source: PLoS One. 2018 Feb 23;13(2):e0192792. doi: 10.1371/journal.pone.0192792 (PMC5825068; doi:10.1371/journal.pone.0192792)
Supplement: S1 Text — (DOCX) [file pone.0192792.s001.docx]

Checklist and descriptions of Supporting files

1. The simulation results are the raw data generated by our C# simulation program, which need data processing techniques (our solution is to use VBA) to fit the format as inputs for our QMS and SIPS algorithms. If the readers require more details in the data processing, please contact the authors for more source codes.

2. The QMS results are the data generated by our MATLAB program (based on the simulation results). They are the source data of some other indicators (e.g., TNI) in the paper.

3. The SIPS results (i.e., the critical paths) can be transformed into the four sets that are defined in the paper: , , and , with simple data processing techniques. If the readers require more details in the data processing, please contact the authors for more source codes.

4. The PDF “Hybrid-GA inputs for all cases” also show the whole PDIA results, which are transformed into the proper format as inputs for our hybrid-GA program (also written in MATLAB). This file is extracted from our MATLAB program. If readers are interested, they are welcome to contact the author for the whole source code as well.

Contact information:

Correspondence author:

Hang-fei Huang

State Key Laboratory of Rail Traffic Control and Safety, Beijing Jiaotong University, Beijing 100044

E-mail address: 14114200@bjtu.edu.cn

Mailing address: State Key Laboratory of Rail Traffic Control and Safety, Beijing Jiaotong University, No.3 ShangYuanCun, Haidian District, Beijing, China.

Tel: +86 15210576684
