# Supplementary material for: Real-time energy-saving metro train rescheduling with primary delay identification
Source: PLoS One. 2018 Feb 23;13(2):e0192792. doi: 10.1371/journal.pone.0192792 (PMC5825068; doi:10.1371/journal.pone.0192792)
Supplement: S4 Dataset — (ZIP) [file pone.0192792.s005.zip › S5 Dataset/Hybird_GA inputs for all cases.pdf]

Hybrid-GA inputs according to the PDIA results

Case a

```
CoIndex = [6,19;7,19;8,19;9,19;10,19;11,19;12,19;13,19;
        6,20;7,20;8,20;9,20;10,20;11,20;12,20;
        6,21;7,21;8,21;9,21;10,21;
        10,18;11,18;12,18;13,18;14,18;
        13,17;14,17;15,17;16,17;
        13,16;14,16;15,16;16,16;17,16;
        16,15;17,15;18,15;19,15;
        17,14;18,14;19,14;20,14;
        19,13;20,13];


Other_index = [6,22;6,23;6,24;6,25;6,26;6,27;6,28;6,29;6,30;6,31;6,32;6,33;6,34;
           7,22;7,23;7,24;7,25;7,26;7,27;7,28;7,29;7,30;7,31;7,32;7,33;7,34;
           8,22;8,23;8,24;8,25;8,26;8,27;8,28;8,29;8,30;8,31;8,32;8,33;8,34;
           9,22;9,23;9,24;9,25;9,26;9,27;9,28;9,29;9,30;9,31;9,32;9,33;9,34;
          10,22;10,23;10,24;10,25;10,26;10,27;10,28;10,29;10,30;10,31;10,32;10,33;10,34;
          11,21;11,22;11,23;11,24;11,25;11,26;11,27;11,28;11,29;11,30;11,31;11,32;11,33;11,34;

12,21;12,22;12,23;12,24;12,25;12,26;12,27;12,28;12,29;12,30;12,31;12,32;12,33;12,34;

13,20;13,21;13,22;13,23;13,24;13,25;13,26;13,27;13,28;13,29;13,30;13,31;13,32;13,33;13,34;

14,19;14,20;14,21;14,22;14,23;14,24;14,25;14,26;14,27;14,28;14,29;14,30;14,31;14,32;14,33;1
4,34;

15,18;15,19;15,20;15,21;15,22;15,23;15,24;15,25;15,26;15,27;15,28;15,29;15,30;15,31;15,32;1
5,33;15,34;

16,18;16,19;16,20;16,21;16,22;16,23;16,24;16,25;16,26;16,27;16,28;16,29;16,30;16,31;16,32;1
6,33;16,34;

17,17;17,18;17,19;17,20;17,21;17,22;17,23;17,24;17,25;17,26;17,27;17,28;17,29;17,30;17,31;1
7,32;17,33;17,34;

18,16;18,17;18,18;18,19;18,20;18,21;18,22;18,23;18,24;18,25;18,26;18,27;18,28;18,29;18,30;1
8,31;18,32;18,33;18,34;
```

19,16;19,17;19,18;19,19;19,20;19,21;19,22;19,23;19,24;19,25;19,26;19,27;19,28;19,29;19,30;19,31;19,32;19,33;19,34;

20,15;20,16;20,17;20,18;20,19;20,20;20,21;20,22;20,23;20,24;20,25;20,26;20,27;20,28;20,29;20,30;20,31;20,32;20,33;20,34];

%Case a
minimum_t=[241,141,147,142,151,140,148,147,224,195,197,205,212,217,219,168,170,166,170,157,154,159,155,156,157,92,96,93,95,89,93,96,91,89,97,96,99,100,117,116,127,115,125,134];
maximum_t=[300,230,230,230,230,230,230,230,310,310,310,310,310,310,310,258,258,258,258,258,236,236,236,236,236,158,158,158,158,160,160,160,160,160,166,166,166,166,198,198,198,198,210,210];

Case b

co=[5,8;6,8;5,9;6,9;
6,11;7,11;8,11;9,11;6,12;7,12;8,12;9,12;
7,16;8,16;9,16;10,16;7,17;8,17;8,17;9,17;
8,20;9,20;10,20;11,20;8,21;9,21;10,21;11,21;
9,12;10,12;11,12;12,12;13,12;9,13;10,13;11,13;12,13;13,13;
10,26;11,26;12,26;13,26;10,27;11,27;12,27;13,27];

other=[5,9;5,10;5,11;5,12;5,13;5,14;5,15;5,16;5,17;5,18;5,19;5,20;5,21;5,22;5,23;5,24;5,25;5,26;5,27;5,28;5,29;5,30;5,31;5,32;5,33;5,34;
6,13;6,14;6,15;6,16;6,17;6,18;6,19;6,20;6,21;6,22;6,23;6,24;6,25;6,26;6,27;6,28;6,29;6,30;6,31;6,32;6,33;6,34;
7,13;7,14;7,15;7,18;7,19;7,20;7,21;7,22;7,23;7,24;7,25;7,26;7,27;7,28;7,29;7,30;7,31;7,32;7,33;7,34;
8,13;8,14;8,15;8,18;8,19;8,22;8,23;8,24;8,25;8,26;8,27;8,28;8,29;8,30;8,31;8,32;8,33;8,34;
9,14;9,15;9,18;9,19;9,22;9,23;9,24;9,25;9,26;9,27;9,28;9,29;9,30;9,31;9,32;9,33;9,34;
10,14;10,15;10,18;10,19;10,22;10,23;10,24;10,25;10,28;10,29;10,30;10,31;10,32;10,33;10,34;
11,14;11,15;11,16;11,17;11,18;11,19;11,22;11,23;11,24;11,25;11,28;11,29;11,30;11,31;11,32;11,33;11,34;
12,14;12,15;12,16;12,17;12,18;12,19;12,20;12,21;12,22;12,23;12,24;12,25;12,28;12,29;12,30;12,31;12,32;12,33;12,34;
13,14;13,15;13,16;13,17;13,18;13,19;13,20;13,21;13,22;13,23;13,24;13,25;13,28;13,29;13,30;13,31;13,32;13,33;13,34];

min=[139,138,201,198,334,344,353,358,158,148,278,277,268,145,139,149,120,119,125,122,374,371,364,368,199,194,198,194,277,272,266,262,256,153,164,165,159,157,413,412,406,379,174,175,182,186];

max=[174,174,270,270,400,400,400,400,192,192,300,300,300,200,200,200,158,158,158,158,410,410,410,410,258,258,258,258,384,384,384,384,384,210,210,210,210,210,500,500,500,500,234,234,234,234];

Case c (in addition to Case b input)

co add: [7,18;8,18;9,18;10,18;11,18;12,18;13,18];

add max [236,236,236,236,236,236,236];
add min [172,179,179,183,183,184,184];
